# The Study of Sensory Aging in Existing Cohorts of Older Adults

**DOI:** 10.1093/geroni/igaa057.2946

**Published:** 2020-12-16

**Authors:** Joshua Ehrlich

## Abstract

Sensory impairments in later life are associated with numerous adverse health outcomes, including falls, depression, cognitive decline and dementia, and loss of independence. While population-based cohort studies offer a wealth of data for studying health and aging, these studies have not been widely leveraged to investigate the epidemiology of sensory impairment and the effect of sensory impairments on health outcomes and well-being in older adults. This symposium will provide attendees with examples from presenters’ own research using sensory function data from large-scale and population-based cohort studies. Presentations will also describe available sensory data and opportunities to improve measures of sensory function in cohort studies. Finally, presenters will focus on methodological considerations when studying the effect of sensory impairment on health outcomes, trends, and trajectories in older adults using data from cohort studies. The symposium will include presentations on vision, hearing, olfaction, and nesting of a randomized controlled trial within an existing cohort study. Participants will gain a clear understanding of the importance of measuring sensory function; methods for integrating sensory measures into existing cohorts; and the original results of analyses that have used sensory data from existing cohorts to study health and well-being in older adults. Sensory Health Interest Group Sponsored Symposium.

